# End-Systolic Elastance and Ventricular-Arterial Coupling Reserve Predict Cardiac Events in Patients with Negative Stress Echocardiography

**DOI:** 10.1155/2013/235194

**Published:** 2013-08-19

**Authors:** Tonino Bombardini, Marco Fabio Costantino, Rosa Sicari, Quirino Ciampi, Lorenza Pratali, Eugenio Picano

**Affiliations:** ^1^Institute of Clinical Physiology, National Research Council, Via Moruzzi 1, 56124 Pisa, Italy; ^2^Cardiology Department, San Carlo Hospital, Via Potito Petrone, 85100 Potenza, Italy; ^3^Division of Cardiology, Fatebenefratelli Hospital, Viale Principe di Napoli 12, 82100 Benevento, Italy

## Abstract

*Background*. A maximal negative stress echo identifies a low-risk subset for coronary events. However, the potentially prognostically relevant information on cardiovascular hemodynamics for heart-failure-related events is unsettled. Aim of this study was to assess the prognostic value of stress-induced variation in cardiovascular hemodynamics in patients with negative stress echocardiography. *Methods*. We enrolled 891 patients (593 males mean age 63 ± 12, ejection fraction 48 ± 17%), with negative (exercise 172, dipyridamole 482, and dobutamine 237) stress echocardiography result. During stress we assessed left ventricular end-systolic elastance index (*E*_LV_I), ventricular arterial coupling (VAC) indexed by the ratio of the *E*_LV_I to arterial elastance index (*E*_a_I), systemic vascular resistance (SVR), and pressure-volume area (PVA). Changes from rest to peak stress (reserve) were tested as predictors of main outcome measures: combined death and heart failure hospitalization. *Results*. During a median followup of 19 months (interquartile range 8–36), 50 deaths and 84 hospitalization occurred. Receiver-operating-characteristic curves identified as best predictors *E*_LV_I reserve for exercise (AUC = 0.871) and dobutamine (AUC = 0.848) and VAC reserve (AUC = 0.696) for dipyridamole. *Conclusions*. Patients with negative stress echocardiography may experience an adverse outcome, which can be identified by assessment of *E*_LV_I reserve and VAC reserve during stress echo.

## 1. Introduction

A maximal negative stress echo identifies a low-risk subset for coronary events. However, prognostically relevant information on cardiovascular hemodynamics for heart-failure-related events is still unsettled. When a physiological (exercise) or pharmacological (dipyridamole, dobutamine) stress echo is scheduled, interest focuses on wall motion segmental contraction abnormalities to diagnose ischemic response to stress [1] and on left ventricular ejection fraction to assess contractile reserve. Echocardiographic evaluation of volumes (plus standard assessment of heart rate and blood pressure) is ideally suited for the quantitative and accurate calculation of a set of parameters allowing a complete characterization of cardiovascular hemodynamics (including cardiac output and systemic vascular resistance), left ventricular elastance (mirroring left ventricular contractility, theoretically independent of preload and afterload changes heavily affecting the ejection fraction), arterial elastance, ventricular arterial coupling (a central determinant of net cardiovascular performance in normal and pathological conditions), and pressure-volume area, an index of LV oxygen consumption [2, 3].

All these parameters, at least in principle, are available in the stress echocardiography laboratory since all of them require the accurate estimation of left ventricular volumes and stroke volume. Aim of the study was to assess the prognostic value of stress-induced variation in cardiovascular hemodynamics in patients with negative stress echocardiography.

## 2. Methods

### 2.1. Patients

**Figure 1:**
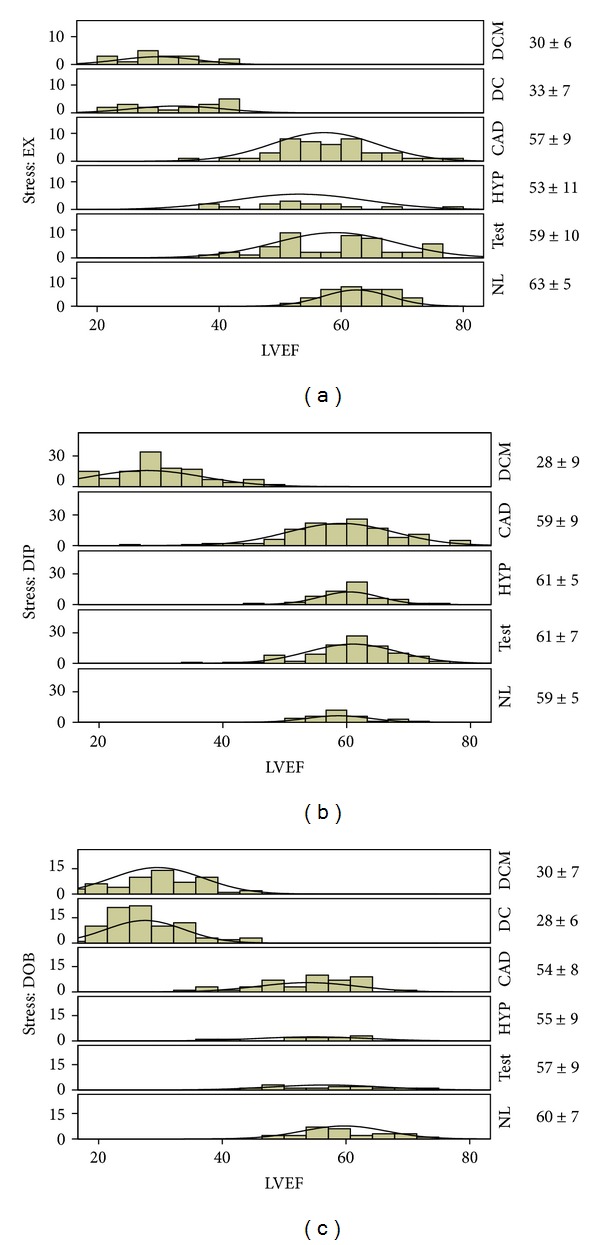
Histogram of ejection fractions in the patient population (a) Exercise stress patients. (b) Dipyridamole stress patients. (c) Dobutamine stress patients. Next to each panel is the mean SD for the LVEF. CAD: coronary artery disease; CI: cardiac Index; DC: dilated ischemic cardiomyopathy; DCM: idiopathic dilated cardiomyopathy; DIP: dipyridamole; DOB: dobutamine; *E*_a_I: arterial elastance index; *E*_LV_I: LV end-systolic elastance index; *E*_LV_I/*E*_a_I: ventricular-arterial coupling ratio; EX: exercise; HYP: hypertensives; NL: normals; PVA: pressure-volume area; SVR: systemic vascular resistance; and TEST: diagnostic tests.

From January 2003 1174 patients underwent stress echocardiography in seven quality-controlled stress echo laboratories (Pisa, Potenza, Benevento, Cesena, Bergamo, and Belgrade). Informed consent was obtained from all patients (or their guardians) before testing, and the study protocol was approved by the institutional ethics committee. Stress echo data were collected and analyzed by stress echocardiographers not involved in patient care. The data of 258 of these patients have been already published regarding aspects related to feasibility [4, 5], diagnostic results [6, 7], and short-term outcome [8]. Exclusion criteria were significant congenital heart disease, unsatisfactory imaging of left ventricle at rest or during stress, atrial fibrillation, positive stress echocardiography, severe mitral regurgitation, or no available followup data. From the initial population of 1174 patients, 118 were excluded for stress echo positivity, 11 for congenital heart disease, 18 for atrial fibrillation, 41 for unsatisfactory echo imaging, and 32 were lost at follow-up; 63 patients with severe stress mitral regurgitation underwent surgical repair; of 118 patients with positive stress, 2 had heart transplants, 40 had percutaneous coronary interventions, 25 coronary artery bypass grafting (8 with left ventricular remodeling, 4 with mitral valve repair), and 51 were followed on medical therapy. Thus, the study population included 891 patients, 593 (67%) men, 298 (33%) women; mean age 63 (SD 12) years, ejection fraction 47 ± 12% (Figure 1), with negative stress echo by wall motion criteria and follow-up data. Patients were categorized ex-post as normals, *n* = 91; idiopathic dilated cardiomyopathy, *n* = 222; known coronary artery disease, *n* = 331 (dilated ischemic cardiomyopathy, *n* = 102; not dilated, *n* = 229); diagnostic tests, *n* = 162, hypertensive, *n* = 85. Diagnostic tests were stress tests in patients with low pretest probability of CAD, ECG abnormalities at rest or exercise electrocardiography, and no LV dilation. The characteristics of the study patients are reported in Table 1. Patients with normal rest and peak normal left ventricular function, no drug therapy, were the normals. Diagnosis of coronary artery disease was based upon history of myocardial infarction or coronary revascularization and/or presence of ≥1 angiographically documented coronary stenosis > 50%. The diagnosis of idiopathic dilated cardiomyopathy was made on the basis of echocardiography (left ventricular dilation and diffuse hypocontractility) and coronary angiography (no significant coronary artery stenosis).

Stress echocardiography was performed on anti-ischemic medical therapy in 510 patients (57%) (Table 1). The stressor used (exercise, dipyridamole, dobutamine) was chosen on the basis of specific contraindications, local facilities, and physician's preferences. Dobutamine was the preferred stressor in case for viability assessment [1]. Figure 1 shows left ventricular ejection fraction values in this patient population. 

### 2.2. Stress Protocol

Two-dimensional echocardiography and 12-lead electrocardiographic monitoring were performed in combination with semisupine bicycle exercise, high-dose (up to 40 *μ*g/kg/min) dobutamine, or high-dose dipyridamole (84 mg/kg/min, over 6 min), according to protocols suggested by the European Association of Echocardiography [1] guidelines. During the procedure, blood pressure and the electrocardiogram were recorded each minute. The test was stopped in cases of obvious, severe inducible wall motion abnormalities, intolerable symptoms, or limiting side effects, including hypertension (systolic blood pressure > 220 mmHg; diastolic blood pressure > 120 mmHg), hypotension (relative or absolute: >30 mmHg decrease in blood pressure), supraventricular arrhythmias (supraventricular tachycardia or atrial fibrillation), ventricular arrhythmias (ventricular tachycardia, frequent, polymorphous premature ventricular beats), and bradyarrhythmias. A maximal test was defined by the achievement of 85% of age-predicted maximal heart rate and maximal drug dose in pharmacological studies.

### 2.3. Echocardiographic Analysis

Echocardiographic images were semiquantitatively assessed using a 17-segment, four-point scale model of the left ventricle [1]. A wall motion score index was derived by dividing the sum of individual segment scores by the number of interpretable segments. Left ventricular ejection fraction was assessed using the biplane Simpson method [9]. Ischemia was defined as stress-induced new wall motion abnormality, or worsening of pre-existing wall motion abnormality, or biphasic response (i.e., low-dose improvement followed by high-dose deterioration). By selection, all patients had negative stress echo by wall motion criteria. Improvement of wall motion score index between resting and peak of stress indicated myocardial viability [10].

### 2.4. Volume Analysis

All patients underwent transthoracic echocardiography at baseline and at peak of stress. After completion of the study, echocardiographic images were read by one experienced cardiologist unaware of the identity of the patient. Left ventricular end-diastolic and end-systolic volumes were measured from apical four- and two-chamber views, using the biplane Simpson method [11]. Only representative cycles with optimal endocardial visualization were measured and the average of three measurements was taken. The endocardial border was traced, excluding the papillary muscles. The frame captured at the R wave of the ECG was considered to be the end-diastolic frame, and the frame with the smallest left ventricular silhouette the end-systolic frame. All cardiac volumes were normalized to body surface area, yielding their respective indexes: end-systolic volume index and stroke volume index. 

### 2.5. Blood Pressure Analysis

One nurse recorded blood pressures at rest and during each individual study. The blood pressure recording was made using a sphygmomanometer and the diaphragm of a standard stethoscope. Systolic blood pressure and diastolic blood pressure was obtained in the right arm (with patient lying in a left rotated supine position during pharmacological stress). Patients were told to let their right hand go limp when blood pressure was measured. End-systolic pressure was approximated as 0.9x brachial systolic blood pressure, a noninvasive estimate of end-systolic pressure that accurately predicts pressure-volume loop measurements of end-systolic pressure [12]. Pulse pressure was calculated as the difference between systolic and diastolic blood pressure and mean arterial pressure as (2 ∗ diastolic blood pressure + systolic blood pressure)/3. The indexes of ventricular and arterial elastance were calculated as (1) left ventricular end-systolic elastance index (*E*_LV_I) = end-systolic pressure/end-systolic volume index, (2) arterial elastance index (*E*_a_I) = end-systolic pressure/stroke volume index, and (3) ventricular-arterial coupling ratio (*E*_LV_I/*E*_a_I) = stroke volume index/end-systolic volume index [13].

The noninvasive assessment of *E*_LV_I is based on the equation: *E*_LV_I = (end-systolic pressure/end-systolic volume index – *V*_0_) and assumes that *V*_0_ (the theoretical volume when no pressure is generated) is negligible compared with end-systolic volume. The *E*_a_I can be calculated as end-systolic pressure/stroke volume. Stroke volume can be readily measured noninvasively (e.g., by echocardiography or gated blood pool scans) [3, 14]. Chen et al. [15] found that the calculation of end-systolic pressure from 0.9x brachial systolic blood pressure reasonably approximated end-systolic pressure measured invasively: the correlation coefficient between the two variables was 0.75, and the regression line had a slope of 1.01 (*P* < 0.0001). From these noninvasive determinations of *E*_LV_I and *E*_a_I, the *E*_LV_I/*E*_a_I ratio can be calculated. The noninvasively obtained values of *E*_LV_I/*E*_a_I closely approximate those obtained invasively [16, 17]. The *E*_LV_I/*E*_a_I is directly related to the ejection fraction [*E*_LV_I/*E*_a_I ≈ 1/((1/EF) − 1)]. The advantage of *E*_LV_I/*E*_a_I over ejection fraction is that examining the components of *E*_LV_I/*E*_a_I allows us to evaluate whether alterations in *E*_LV_I/*E*_a_I are due to alterations in left ventricular properties, arterial properties, or both [18, 19]. Stroke work index (SWI) was calculated as stroke volume index × end-systolic pressure [20]. Pressure-volume area (PVA), an index of left ventricular oxygen consumption [21] was calculated as SWI + potential energy (defined as end-systolic pressure × (end-systolic volume index −*V*_0_)/2) [13], wherein *V*_0_, the volume-axis intercept of the end-systolic pressure volume relationship, was assumed to be zero, as previously reported [22]. Systemic vascular resistance (SVR) was calculated as mean arterial pressure/cardiac output × 80. The stroke volume (mL) was calculated as end-diastolic volume − end-systolic volume. Stroke volume was indexed by dividing it by body surface area. Stroke volume index (mL/m^2^) = stroke volume/body surface area. The Cardiac index (L/min/m^2^) was calculated as: heart rate ∗ stroke volume index. Reserve was defined as the difference in these variables between rest and peak stress.

### 2.6. Followup

All-cause mortality was determined by review of death certificates. Death was considered to be due to cardiovascular causes if the death certificate listed acute myocardial infarction, congestive heart failure, or arrhythmia as the primary cause of death. Sudden death, defined as death occurring unexpectedly within 1 h of the onset of symptoms, was also considered cardiovascular. In order to avoid misclassification of the cause of death, overall mortality was considered. Hospitalization for heart failure was defined as a minimum 1-night hospital stay for a clinical syndrome comprising at least two of the following: paroxysmal nocturnal dyspnea, orthopnea, elevated jugular venous pressure, pulmonary rales, third heart sound, and cardiomegaly or pulmonary edema on chest roentgenography. These clinical signs and symptoms must have represented a clear change from the baseline clinical status of the participant and must have been accompanied by either failing cardiac output as determined by peripheral hypoperfusion (in the absence of other causes such as sepsis or dehydration) or peripheral or pulmonary edema requiring intravenous diuretics, inotropes, or vasodilators.

### 2.7. Statistical Analysis

SPSS 13 for Windows was used for statistical analysis. The statistical analyses included descriptive statistics (frequency and percentage of categorical variables and mean and standard deviation of continuous variables). Pearson chi-square with Fisher's exact test for categorical variables and the Mann-Whitney test for continuous variables for intergroup comparisons were performed to confirm significance (using the Monte Carlo method for small sample comparisons). One-way ANOVA was used to compare continuous variables between groups; when homogeneity of variance was not present, the Kruskal-Wallis test for nonparametric independent samples was used. Our significance tests were two sided, in the sense that sufficiently large departures from the null hypothesis, in either direction, were judged significant if *P* < 0.05. Predefined primary cardiovascular events were defined as the composite of death and heart failure hospitalization. Only the first event was taken into account. Receiver-operator characteristic (ROC) curves were constructed to assess the accuracy of cardiovascular hemodynamic values changes (Δ = reserve) from rest to peak stress to predict primary cardiovascular events. The ROC curve was considered statistically significant if the AUC differed from 0.5, as determined by the *z*-test. The optimal cut point values from the ROC curves were chosen by use of the Youden index.

## 3. Results and Discussion

All studies have been performed by an experienced cardiologist with documented experience in stress echocardiography and who passed the quality-control procedures of stress echocardiography reading according to criteria adopted in the EPIC (Echo Persantine International Cooperative) and in the EDIC (Echo Dobutamine International Cooperative) multicentre studies [10]. By selection of 2D measurements of LV volumes were feasible in all patients. In sixty randomly selected patients there was an excellent interobserver agreement with the Bland Altman method with mean ± SD for LV end-diastolic volume at rest (2.3 ± 18 mL; 95% CI: −38 mL to 34 mL) and at peak stress (5.8 ± 16 mL; 95% CI: −38 mL to 26 mL), LV end-systolic volume at rest (3.6 ± 23 mL; 95% CI: −48 to 41 mL) and at peak stress (0.3 ± 13 mL; 95% CI: −27 to 27 mL). The variability was lower for LV end-diastolic and end-systolic volumes both for pharmacological and exercise echo at low heart rates (<100 bpm) at peak stress. In a subset of study patients (*n* = 17) the intra-observer variability was calculated. The analyses of observer 1 were compared with those previously calculated after a minimum interval of 8 weeks. In this subgroup the agreement was excellent with the Bland Altman method with a mean ± SD for LV end-diastolic volume at rest (4.7 ± 11 mL; 95% CI: −27 mL to 17 mL) and at peak stress (5 ± 15 mL; 95% CI: −34 mL to 24 mL), LV end-systolic volume at rest (3.4 ± 7 mL; 95% CI: −17 to 10 mL) and at peak stress (1.2 ± 7.7 mL; 95% CI: −16.2 to 13.9 mL). By selection no test was interrupted because of limiting side effects, and no test was positive for regional wall motion abnormalities. The main clinical and echocardiographic findings of the study population are shown in Table 1. Figure 1 shows histograms of ejection fractions in this patient population. The mean LVEF increased from 61% ±6% to 72% ±7% in normal subjects and from 46% ±17% to 54% ±18% in patients. Differences between resting and stress echocardiographic variables for different stresses are shown in Table 2. Of 420 subjects with resting wall motion abnormalities, 219 (52%) showed improvement of wall motion abnormalities under stress (wall motion score index rest = 2.23 ± 0.39, versus stress = 1.81 ± 0.46). 

### 3.1. Arterial-Ventricular Coupling Ratio and Its Components at Rest

A reduced end-systolic elastance index (*E*_LV_I) and ventricular-arterial coupling ratio (*E*_LV_I/*E*_a_I) were found in dilated ischemic and idiopathic dilated cardiomyopathy patients (Table 1). Arterial elastance index (*E*_a_I) was significantly higher in idiopathic dilated cardiomyopathy patients. The SVR was similar between patients at rest. The pressure-volume area, an index of oxygen consumption, was higher in hypertensive, dilated ischemic and idiopathic dilated cardiomyopathy patients versus normals.

### 3.2. Comparison of the Coupling Ratio Reserve and Its Components between Groups and Different Stresses

**Figure 2:**
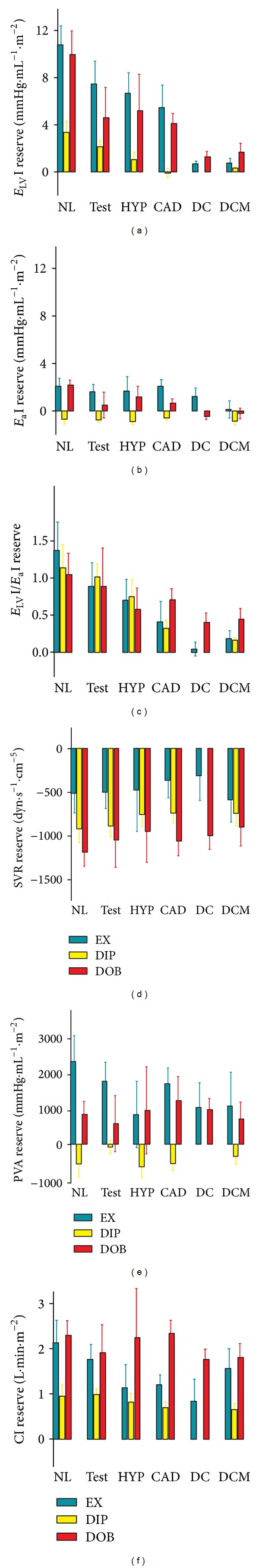
Ventricular-arterial coupling reserve, its components and hemodynamic changes during exercise, dipyridamole and dobutamine stress echocardiographies. Bars show changes form rest to peak stress (reserve) in the patients who underwent exercise (green bars), dipyridamole (yellow bars) and dobutamine (red bars) stress echocardiography. CAD: coronary artery disease; CI: cardiac Index; DC: dilated ischemic cardiomyopathy; DCM: idiopathic dilated cardiomyopathy; *E*_a_I: arterial elastance index; *E*_LV_I: LV end-systolic elastance index; *E*_LV_I/*E*_a_I: ventricular-arterial coupling ratio; HYP: hypertensive; NL: normals; PVA: pressure-volume area; SVR: systemic vascular resistance; and TEST: diagnostic tests.

**Figure 3:**
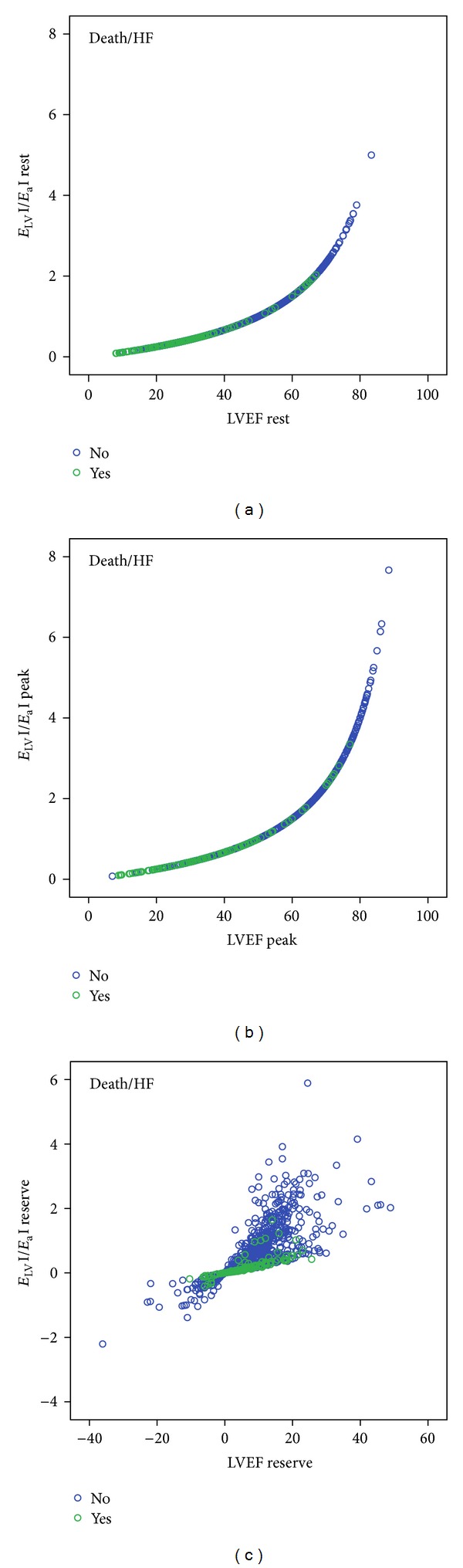
Scatter plot relating ventricular-arterial coupling ratio (*E*_LV_I/*E*_a_I) and left-ventricular ejection fraction (LVEF). (a) Resting values; (b) peak stress values; (c) *E*_LV_ *I*/*E*_a_I and LVEF reserve values. Green circles: patients with follow-up events. Blue circles: patients without follow-up events. *E*_a_I: arterial elastance index; *E*_LV_I: LV end-systolic elastance index; *E*_LV_I/*E*_a_I: ventricular-arterial coupling ratio.

The end-systolic elastance index (*E*_LV_I) increased from 7.1 ± 2.4 to 15 ± 6.6 mmHg·mL^−1^·m^−2^ at peak stress in normal subjects but increased less in the patients (from 4.6 ± 3.4 to 6.6 ± 5.7 mmHg·mL^−1^·m^−2^, *P* < 0.01 versus normals), although the response was heterogeneous at the individual level and between stress types (Figure 2 and Table 2). Thus, there was a twofold difference in the peak stress LV inotropic state, and an even greater difference in the stress induced increase in the inotropic state between normal subjects versus DC and DCM patients (*P* < 0.01). During stress, arterial elastance mildly decreased in the patients (−0.2 ± 1.8 mmHg·mL^−1^·m^−2^) and increased in normal subjects (+1.1 ± 2 mmHg·mL^−1^·m^−2^), *P* < 0.01 between groups. The *E*_LV_I/*E*_a_I reserve during stress was blunted (*P* < 0.05) in dilated ischemic and idiopathic dilated cardiomyopathy patients compared with other groups, with intermediate changes for coronary artery disease patients (Figures 2(a), 2(b), and 2(c)). In exercise and dobutamine tests this was almost entirely due to a blunted increase (*P* < 0.05) in *E*_LV_I from rest to peak stress; interestingly, despite a blunted increase in end-systolic elastance index (*E*_LV_I) in all dipyridamole tests, still coupling (*E*_LV_I/*E*_a_I) reserve was present in normals for a negative arterial elastance (*E*_a_I) reserve in this vasodilator test. The pressure-volume area (PVA) reserve, an index of left ventricular oxygen consumption [3, 18, 21, 23], was negative in dipyridamole tests, while it increased in exercise and dobutamine tests; this is according to the heart rate × systolic blood pressure product, which increased much more in exercise and dobutamine tests versus dipyridamole (Table 2). In accord with their known pharmacological effects, dobutamine and dipyridamole tests induced a greater reduction in SVR from rest to peak exercise than exercise tests (Figures 2(d), 2(e), and 2(f)) [1, 24]. Obviously, there was a complete fit between ventricular-arterial coupling ratio (*E*_LV_I/*E*_a_I) and left ventricular ejection fraction at rest (*R* square cubic = 0.996, Figure 3(a)) at peak stress (*R* square cubic = 0.989, Figure 3(b)). A lower fit exists between ventricular-arterial coupling ratio (*E*_LV_I/*E*_a_I) reserve and ejection fraction reserve (*R* square linear = 0.561, Figure 3(c)). Compared with exercise and dobutamine tests, patients undergoing dipyridamole had a lower peak stress cardiac index (dipyridamole = 2.8 ± 1.1 L·min·m^−2^ versus exercise = 3.7 ± 1.2 L·min·m^−2^, versus dobutamine = 4 ± 1.3 L·min·m^−2^, *P* < 0.01).

### 3.3. Followup Data

**Figure 4:**
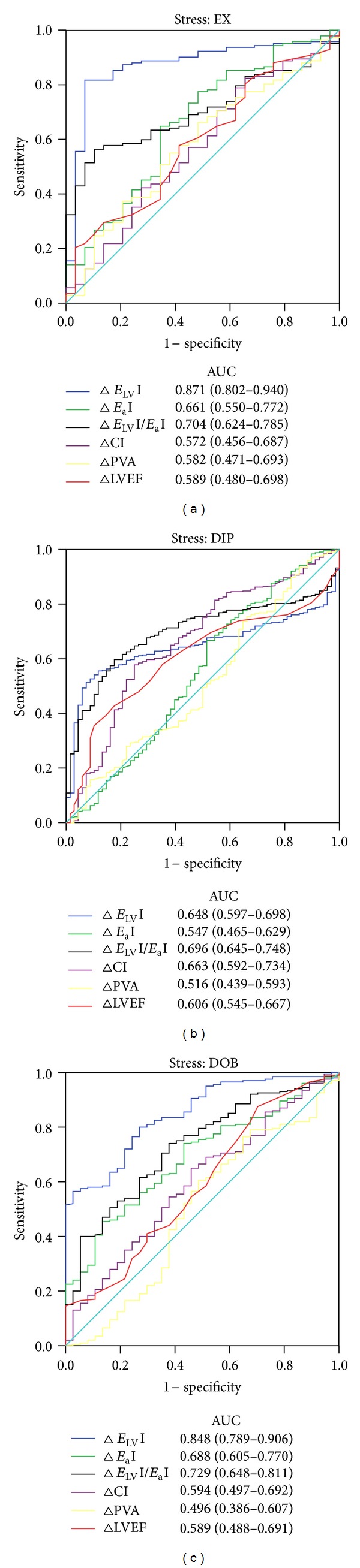
Receiver-operating-characteristic curves and the corresponding areas under the curve (AUC) shows the predictor performance of ventricular-arterial coupling reserve, its components and hemodynamic changes (Δ) during stress in the exercise (EX), dipyridamole (DIP), and dobutamine (DOB) subsets. CI: cardiac Index; *E*_a_I: arterial elastance index; *E*_LV_I: LV end-systolic elastance index; *E*_LV_I/*E*_a_I: ventricular-arterial coupling ratio; LVEF: left ventricular ejection fraction; and PVA: pressure-volume area.

During a median followup of 19 months (interquartile range 8–36), 50 deaths and 84 hospitalizations for heart failure occurred. Of the 84 patients with hospitalizations for heart failure, 3 underwent heart transplant and 27 a cardiac resychronization therapy defibrillator implant. According to physiopathological data, the event rate was higher in dilated ischemic and idiopathic dilated cardiomyopathy (Table 1). Changes from rest to peak stress (Δ values = reserve) were tested as predictors of main outcome measures: combined death and heart failure hospitalization. Receiver-operating-characteristic curves and the corresponding areas under the curve show the predictor performance of hemodynamic changes during stress in the exercise, dipyridamole and dobutamine subsets (Figure 4). The optimal cut point values from the receiver-operator characteristic curves were chosen by use of the Youden index. In the whole group of patients a cut point value for *E*_LV_I reserve of 0.65 mmHg·mL^−1^·m^−2^ predicted events with a sensitivity of 64% and a specificity of 79% (area under the curve = 0.72, 95% CI: 0.684 to 0.756; *P* = 0.000, Youden index = 1.44). In the whole group of patients a cut point value for *E*_LV_I/*E*_a_I reserve of 0.35 predicted events with a sensitivity of 55% and a specificity of 83%, Youden index = 1.38 (area under the curve = 0.71, 95% CI: 0.671 to 0.748; *P* = 0.000).

### 3.4. Comparison of the Coupling Ratio Reserve and Its Components as Prognostic Predictors between Different Stresses

**Figure 5:**
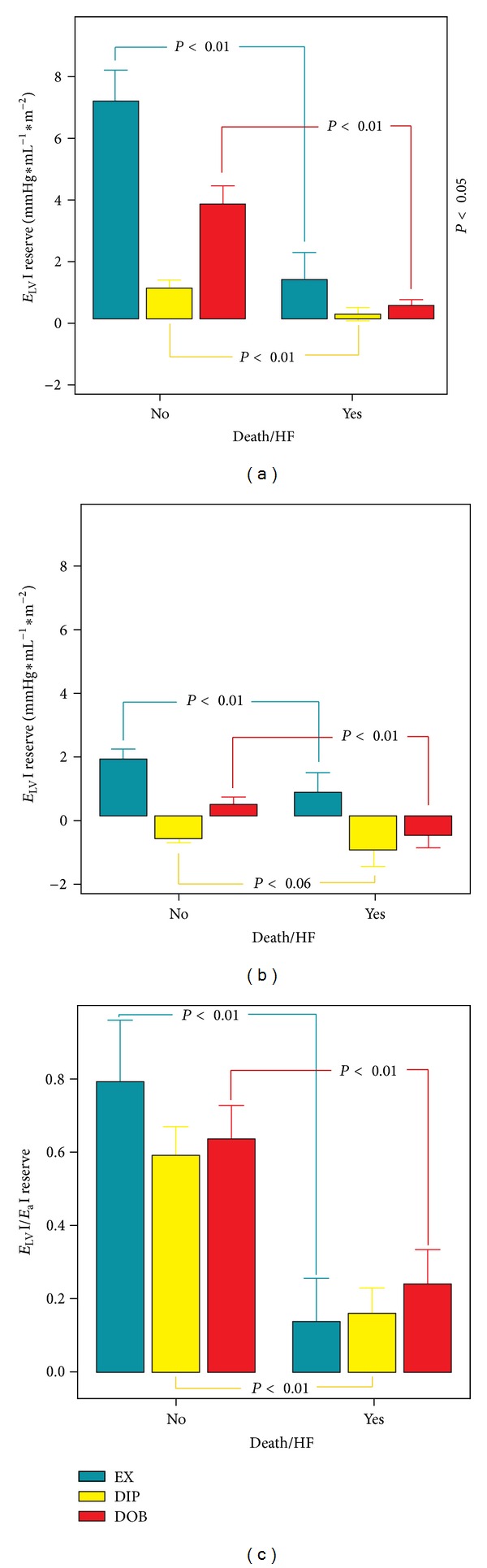
Ventricular-arterial coupling reserve, its components in patients with versus without follow-up events. Bars show changes from rest to peak stress (reserve) in the patients who underwent exercise (green bars), dipyridamole (yellow bars) and dobutamine (red bars) stress echocardiographies. (a) *E*_LV_I: LV end-systolic elastance index reserve; (b) *E*_a_I: arterial elastance index reserve; and (c) *E*_LV_I/*E*_a_I: ventricular-arterial coupling ratio reserve.

The *E*_LV_I/*E*_a_I reserve was smaller (*P* < 0.01) in patients with events, compared to patients without events for all three stress modalities (Figure 5(c)). This was almost entirely due to a smaller increase (*P* < 0.01) in *E*_LV_I from rest to peak exercise in exercise and dobutamine stress (Figure 5(a)); interestingly, despite a blunted increase in contractility in dipyridamole tests, coupling differences were still present for a greater negative arterial elastance reserve in dipyridamole group patients with follow-up events. Despite higher peak *E*_LV_I mean values for exercise and dobutamine versus dipyridamole, patients who experienced events in the followup had similar flat contractile and ventricular-arterial coupling reserve. The optimal *E*_LV_I reserve cutoff was 1.34 mmHg·mL^−1^·m^−2^ for exercise, 0.46 mmHg·mL^−1^·m^−2^ for dipyridamole, and 0.56 mmHg·mL^−1^·m^−2^ for dobutamine tests. The optimal *E*_LV_I/*E*_a_I reserve cutoff was 0.39 for exercise, 0.26 for dipyridamole tests, and 0.22 for dobutamine tests (Table 3 and Figure 4). 

Patients with negative stress echocardiography may experience an adverse outcome, which can be identified by end-systolic elastance index (*E*_LV_I) reserve and ventricular-arterial coupling ratio (*E*_LV_I/*E*_a_I) reserve. These data confirm and expand previous observations, suggesting the additional value of these relatively novel indexes over regional wall motion analysis [4, 7, 8, 25]. At rest, in healthy individuals, ventricular-arterial coupling is maintained within a narrow range, which allows the cardiovascular system to optimize energy efficiency at the expense of mechanical efficacy. During stress, an acute mismatch between the ventricular and arterial systems occurs, due to a disproportionate increase in ventricular systolic elastance index *E*_LV_I (versus arterial elastance *E*_a_I), to ensure that sufficient cardiac performance is achieved to meet the increased energetic requirements of the body. As a result ventricular-arterial coupling (*E*_LV_I/*E*_a_I) increased in normals from an average of 1.6 to 3.1 in exercise, to 2.6 in dipyridamole, to 2.6 in dobutamine stress echos. The end-systolic elastance index (*E*_LV_I) reserve and ventricular-arterial coupling ratio (*E*_LV_I/*E*_a_I) reserve were markedly lower at peak stress in dilated ischemic and idiopathic dilated cardiomyopathy patients (Figure 2). Obviously follow-up event frequency was higher in more severely diseased groups. However, an adverse outcome could be still identified by *E*_LV_I reserve and *E*_LV_I/*E*_a_I reserve (Figure 4). The *E*_LV_I/*E*_a_I reserve was nearly three times less in patients with versus without follow-up events, in the exercise, dipyridamole, and dobutamine groups (Figure 5). Several papers linked contractile reserve to prognosis, demonstrating more follow-up events in the presence of blunted contractile reserve [4, 7, 8, 25–28]. But longitudinal studies to evaluate whether alterations in *E*_LV_I/*E*_a_I reserve provide any prognostic information for adverse outcomes, such as heart failure, are lacking [3]. At the experimental level some reports link acute heart failure and dilated ischemic cardiomyopathy to altered ventricular-arterial coupling ratio. In tachycardia-induced heart failure in anesthetized dogs [29] a coupling defects occurred early prior to significant pump dysfunction. In dilated ischemic cardiomyopathy in rats characterized by infarct expansion, ventricular-arterial coupling progressively deteriorated [30]. For humans, at rest [31], 41 patients with previous myocardial infarction were enrolled. Ventricular-arterial coupling assessed by echocardiography demonstrated good accuracy in predicting long-term cardiovascular mortality comparable with that of BNP. Our data demonstrated that patients with negative stress echocardiography may experience an adverse outcome, which can be identified by end-systolic elastance index (*E*_LV_I) and ventricular-arterial coupling ratio (*E*_LV_I/*E*_a_I) reserve. Our work adds two clinically relevant pieces of information as follows:

1. *E*_LV_I and *E*_LV_I/*E*_a_I reserve during exercise, dipyridamole and dobutamine stress echocardiographies clearly outperforms ejection fraction reserve (Figure 4).
2. A prognostically relevant cutoff value of *E*_LV_I and *E*_LV_I/*E*_a_I reserve *can be assessed*, regardless of the stress employed (Figure 5).

### 3.5. Limitations

Some of the methodological issues pertaining to the noninvasive assessment of ventricular-arterial coupling ratio (*E*_LV_I/*E*_a_I) and its components during stress echocardiography should be highlighted [3]. The noninvasive measurement of end-systolic elastance index (*E*_LV_I) from end-systolic pressure/end-systolic volume index ratio has its own limitations. (1) It assumes that *V*_0_ (the theoretical volume when no pressure is generated) is negligible compared with end-systolic volume; *V*_0_ has not been well characterized in humans, particularly during exercise. In healthy adult dogs, Little and Cheng [32] found that although the absolute values of *V*_0_ did not significantly change during exercise, *V*_0_ as a percentage of end-systolic volume increased by 9%. In contrast, in healthy subjects, Starling [21] found that *V*_0_, both in absolute values and as a percentage of end-systolic volume, did not appreciably change during dobutamine infusion. (2) The formula used to noninvasively estimate end-systolic pressure (end-systolic pressure = 0.9 ∗ systolic blood pressure) has not been validated during exercise. In this regard, methodologies that use radial applanation tonometry may be of help as they allow noninvasive and accurate estimations of central systolic blood pressure at rest and during exercise, at least in the supine position and at low intensities of exercise [33]. However, we should emphasize that the ventricular-arterial coupling ratio is not affected by central pressures because the pressure terms in the numerator and the denominator are canceled out, and the noninvasive values of *E*_LV_I/*E*_a_I can be regarded as relatively accurate. On the other hand, the values of *E*_LV_I and *E*_a_I should be viewed as approximations. Blood pressure measurements are simpler and more accurate during pharmacological stress echocardiography (dipyridamole or dobutamine) since no movement-related artifacts can occur [1]. Also, volume measurement is simpler during pharmacological stress echocardiography, with the patient lying down on the left side, for an optimal visualization of the cardiac structures, especially during dipyridamole stress echo, due to the low heart rate values at peak stress [34].

*Wall Motion Score Index and Stress*. 41 patients were excluded for unsatisfactory echo imaging. We previously demonstrated in 73 patients undergoing stress echocardiography that of all the potential 1241 segments, 1214 (97.8%) were visualized at rest and 1146 (92.3%) at peak stress. The inter-observer agreement for 2D echo was good at rest (*κ* value = 0.75) and at peak stress (*κ* value = 0.70). The agreement between the two observers was further evaluated by separating the total population according to image quality at peak stress and patient decubitus during the stress (semi-supine decubitus for exercise and left lateral decubitus for pharmacological stressors). In the presence of a bad quality image the agreement between the two observers decreases significantly in the case of semi-supine decubitus (*κ* value = 0.88 with good quality images and 0.69 with bad quality images). Moreover, when patients reached high heart rates, agreement between the two observers significantly decreased both in left lateral and semi-supine decubitus (*κ* value: pharmacological stress heart rate < 100 bpm = 0.83; heart rate ≥ 100 bpm = 0.49; exercise stress heart rate < 100 bpm = 0.88; and heart rate ≥ 100 bpm = 0.78) [35]. 

## 4. Conclusions

Patients with negative stress echocardiography may experience an adverse outcome, which can be identified by end-systolic elastance index and ventricular-arterial coupling ratio reserve. 

## Figures and Tables

**Table 1:** Demographics, resting values, and follow-up events.

*n* (%) or mean ± SD	NL	TEST	HYP	CAD	DC	DCM
Patients	91	162	85	229	102	222
Age (years)	56 ± 15	62 ± 12	66 ± 11	66 ± 10	67 ± 9	61 ± 12*
Males	52 (57)	73 (45)	47 (55)	173 (76)	87 (85)	161 (73)^*χ*^
Body surface area (m^2^)	1.84 ± 0.19	1.81 ± 0.18	1.84 ± 0.20	1.87 ± 0.19	1.82 ± 0.185	1.85 ± .20
Previous myocardial infarction	— (0)	— (0)	— (0)	111 (49)	102 (100)	— (0)^*χ*^
Beta blockers on	— (0)	53 (33)	42 (49)	119 (52)	54 (53)	133 (60)^*χ*^
Angiotensin converting enzyme inhibitor on	— (0)	36 (22)	45 (53)	87 (38)	82 (80)	183 (82)
Calcium blockers on	— (0)	31 (19)	23 (27)	55 (24)	2 (2)	7 (3)^*χ*^
Wall motion score index	1 ± 0	1.03 ± 0.15	1.06 ± 0.23	1.17 ± 0.3	2.26 ± 0.37	2.23 ± 0.34*
LV ejection fraction (%)	60 ± 6	60 ± 8	58 ± 7	58 ± 9	28 ± 7	28 ± 9*
*E*_LV_I/*E*_a_I (ratio)	1.6 ± 0.4	1.6 ± 0.6	1.5 ± 0.4	1.5 ± 0.6	0.4 ± 0.1	0.4 ± 0.2*
*E*_LV_I (mmHg · mL^−1^ · m^−2^)	7.1 ± 2.4	7 ± 3.6	6.3 ± 2.8	6.2 ± 3	1.7 ± 0.7	1.9 ± 1*
*E*_a_I (mmHg · mL^−1^ · m^−2^)	4.5 ± 1.3	4.4 ± 1.5	4.3 ± 1.5	4.3 ± 1.5	4.2 ± 1.4	5.4 ± 3.8*
SWI (mmHg · mL · m^−2^)	3,248 ± 1,020	3,626 ± 1,187	4,456 ± 1,473	3,819 ± 1,254	2,817 ± 942	2,861 ± 1,437*
PVA (mmHg · mL · m^−2^)	4,306 ± 1,276	4,845 ± 1,483	6,052 ± 1,858	5,275 ± 1,722	6,493 ± 2,147	6,414 ± 2,737*
LV end-systolic volume index (mL/m^2^)	18 ± 5	20 ± 8	24 ± 9	24 ± 10	71 ± 26	67 ± 30*
Stroke volume index (mL/m^2^)	28 ± 7	30 ± 9	34 ± 10	31 ± 9	27 ± 8	27 ± 12*
LV end-diastolic volume index (mL/m^2^)	46 ± 11	50 ± 14	58 ± 17	54 ± 16	99 ± 31	94 ± 38*
End-systolic pressure (mmHg)	117 ± 17	121 ± 19	133 ± 19	123 ± 18	104 ± 18	107 ± 19*
Heart rate at rest (bpm)	70 ± 13	70 ± 13	71 ± 14	69 ± 12	73 ± 14	76 ± 16*
Cardiac index (L · min · m^−2^)	1.933 ± 560	2.089 ± 624	2.364 ± 735	2.052 ± 659	1.991 ± 743	1.971 ± 989*
SVR (dyn · s^−1^ · cm^−5^)	2,205 ± 698	2,151 ± 816	2,063 ± 712	2,130 ± 750	2,129 ± 825	2,390 ± 1424
Follow-up events, *n* (%)	— (0)	7 (4)	— (0)	12 (5)	30 (29)	85 (38)^*χ*^
Death	— (0)	4 (3)	— (0)	2 (1)	8 (8)	36 (16)^*χ*^
Heart failure	— (0)	3 (2)	— (0)	10 (4)	22 (22)	49 (22)^*χ*^

*P < 0.01 between groups (ANOVA); ^*χ*^chi square *P* < 0.01 between groups.

CAD: coronary artery disease; CI: cardiac index; DC: dilated ischemic cardiomyopathy; DCM: idiopathic dilated cardiomyopathy; *E*_a_I: arterial elastance index; *E*_LV_I: LV end-systolic elastance index; *E*_LV_I/*E*_a_I: ventricular-arterial coupling ratio; HYP: hypertensives; NLs: normals; PVA: pressure-volume area; SVR: systemic vascular resistance; and TEST: diagnostic tests.

**Table 2:** Stress echocardiography induced variation in cardiovascular hemodynamics.

*n* (%) or mean ± SD	Exercise stress echo	Dipyridamole stress echo	Dobutamine stress echo
Patients	172 (19%)	482 (54%)	237 (27%)
Age, years	59 ± 13^§^	63 ± 12	66 ± 10
Males	133 (77%)	287 (60%)*	173 (73%)
Body mass index (kg/m^2^)	1.87 ± 0.18^‡^	1.85 ± 0.2*	1.81 ± 0.18
Left ventricular ejection fraction (%)			
Rest	53 ± 14^‡^	50 ± 17*	39 ± 15
Peak stress	60 ± 17^‡^	57 ± 18*	51 ± 17
Reserve	7 ± 9^‡^	7 ± 8*	12 ± 9
Heart rate × systolic blood pressure			
Rest	9,546 ± 2,461^‡^	9,579 ± 2,393*	8,253 ± 2,058
Peak stress	22,652 ± 6,430^§^	11,035 ± 2,749*	18,027 ± 6,118
Reserve	13,105 ± 5,875^§^	1,455 ± 2,131*	9,773 ± 6,219
*E*_LV_I/*E*_a_I			
Rest	1.33 ± 0.70^‡^	1.23 ± 0.70*	0.79 ± 0.56
Peak stress	2 ± 1.34^‡^	1.76 ± 1.15*	1.36 ± 0.98
Reserve	0.67 ± 0.96	0.53 ± 0.78	0.57 ± 0.62
*E*_LV_I, mmHg·mL^−1^·m^−2^			
Rest	5.86 ± 4.21^‡^	5.37 ± 3.21*	3.17 ± 2.42
Peak stress	12 ± 9.1^§^	6.28 ± 4.17	6.46 ± 6.12
Reserve	6.18 ± 6.1^§^	0.91 ± 2.46*	3.29 ± 4.17
*E*_a_I, mmHg·mL^−1^·m^−2^			
Rest	4.28 ± 1.39^¶^	4.97 ± 2.82*	4.13 ± 1.5
Peak stress	6.1 ± 2.7^§^	4.19 ± 2.56	4.35 ± 1.76
Reserve	1.65 ± 1.95^§^	−0.78 ± 1.42*	0.22 ± 1.62
SWI, mmHg·mL^−1^·m^−2^			
Rest	3,465 ± 1,156^‡^	3,594 ± 1,457*	3,082 ± 1,235
Peak stress	4,963 ± 1,705^§^	3,624 ± 1,440*	4,274 ± 1,665
Reserve	1,498 ± 1,607^¶^	30 ± 1,035*	1,192 ± 1,330
PVA, mmHg·mL^−1^·m^−2^			
Rest	5,248 ± 1,947^‡^	5,971 ± 2,159	5,844 ± 2,234
Peak stress	6,852 ± 2,209^‡^	5,202 ± 2,018*	6,728 ± 2,598
Reserve	1,604 ± 1,790^§^	−395 ± 1,226*	884 ± 1,769
Stroke volume index (mL/m^2^)			
Rest	29.6 ± 8.6	29.3 ± 10.6	28.6 ± 9.5
Peak stress	30.2 ± 8.7^‡^	32.3 ± 11.6	32.9 ± 10.2
Reserve	0.6 ± 9.6^§^	3.1 ± 7.5	4.4 ± 9.1
End-systolic pressure (mmHg)			
Rest	117 ± 19^§^	122 ± 20*	107 ± 18
Peak stress	165 ± 30^§^	112 ± 19*	130 ± 28
Reserve	47 ± 25^§^	−10 ± 15*	23 ± 25
Heart rate (bpm)			
Rest	73 ± 14	71 ± 14	70 ± 14
Peak stress	122 ± 21^¶^	89 ± 16*	123 ± 24
Reserve	49 ± 19^¶^	18 ± 13*	53 ± 28
Cardiac index (L·min·m^−2^)			
Rest	2.152 ± 704	2.044 ± 789	1.985 ± 746
Peak stress	3.693 ± 1.246^§^	2.847 ± 1.106*	4.008 ± 1.338
Reserve	1.540 ± 1.153^§^	803 ± 795*	2.023 ± 1.101
SVR (dyn·s^−1^·cm^−5^)			
Rest	1,972 ± 696^¶^	2,287 ± 1,113	2,187 ± 820
Peak stress	1,576 ± 880^‡^	1,524 ± 911*	1,213 ± 481
Reserve	−442 ± 619^§^	−763 ± 668*	−974 ± 652

^§^Significant differences between exercise and both dipyridamole and dobutamine patients; ^‡^significant differences between exercise and dobutamine pts; *significant differences between dipyridamole and dobutamine patients;  ^¶^significant differences between exercise and dipyridamole patients.

*E*_LV_I/*E*_a_I: ventricular-arterial coupling ratio; *E*_a_I: effective arterial elastance; *E*_LV_I: left ventricular end-systolic elastance; PVA: pressure-volume area; SVR: systemic vascular resistance; and SWI: stroke work index.

**Table 3:** Prognostic value of stress-induced variation in cardiovascular hemodynamics in patients with negative stress echocardiography.

	AUC	95% CI	*P* =	Cut point	Sensitivity	Specificity	Youden index
Exercise stress echo							
*E*_ LV_I reserve, mmHg · mL^−1^ · m^−2^	0.871	0.802–0.940	0.000	1.34	86%	83%	1.69
*E*_a_I, reserve, mmHg · mL^−1^ · m^−2^	0.661	0.550–0.772	0.007	1	65%	66%	1.3
*E*_ LV_I/*E*_a_I reserve	0.704	0.624–0.785	0.000	0.39	57%	90%	1.46
Cardiac index reserve, L · min · m^−2^	0.572	0.456–0.687	0.200	—	—	—	—
PVA reserve, mmHg · mL^−1^ · m^−2^	0.582	0.471–0.693	0.145	—	—	—	—
LV ejection fraction reserve %	0.589	0.480–0.696	0.084	—	—	—	—
SVR reserve, dyn · s^−1^ · cm^−5^	0.440	0.325–0.555	0.307	—	—	—	—

Dipyridamole stress echo							
*E*_ LV_I reserve, mmHg · mL^−1^ · m^−2^	0.648	0.597–0.698	0.000	0.46	54%	90%	1.44
*E*_a_I, reserve mmHg · mL^−1^ · m^−2^	0.547	0.465–0.629	0.213	—	—	—	—
*E*_ LV_I/*E*_a_I reserve	0.696	0.645–0.748	0.000	0.26	60%	82%	1.42
Cardiac index reserve, (L · min · m^−2^)	0.663	0.592–0.734	0.000	0.657	58%	75%	1.33
PVA reserve, mmHg · mL^−1^ · m^−2^	0.516	0.439–0.593	0.677	—	—	—	—
LV ejection fraction reserve (%)	0.606	0.545–0.667	0.003	9%	41%	85%	1.26
SVR reserve, (dyn · s^−1^ · cm^−5^)	0.452	0.367–0.537	0.204	—	—	—	—

Dobutamine stress echo							
*E*_ LV_I reserve mmHg · mL^−1^ · m^−2^	0.848	0.789–0.906	0.000	0.56	80%	73%	1.53
*E*_a_I, reserve mmHg · mL^−1^ · m^−2^	0.688	0.605–0.770	0.000	0.56	46%	86%	1.32
*E*_ LV_I/*E*_a_I reserve	0.729	0.648–0.811	0.000	0.22	74	62	1.36
Cardiac index reserve, (L · min · m^−2^)	0.594	0.497–0.692	0.062	—	—	—	—
PVA reserve, mmHg · mL^−1^ · m^−2^	0.496	0.386–0.607	0.984	—	—	—	—
LV ejection fraction reserve (%)	0.589	0.488–0.691	0.080	—	—	—	—
SVR Reserve, (dyn · s^−1^ · cm^−5^)	0.416	0.308–0.525	0.107	—	—	—	—

*E*_ LV_I/*E*_a_I: ventricular-arterial coupling ratio; *E*_a_I: effective arterial elastance; *E*_LV_I: left ventricular end-systolic elastance; PVA: pressure-volume area; and SVR: systemic vascular resistance.
